# Disability Adjusted Life Years (DALYs) in Terms of Years of Life Lost (YLL) Due to Premature Adult Mortalities and Postneonatal Infant Mortalities Attributed to PM_2.5_ and PM_10_ Exposures in Kuwait

**DOI:** 10.3390/ijerph15112609

**Published:** 2018-11-21

**Authors:** Ali Al-Hemoud, Janvier Gasana, Abdullah N. Al-Dabbous, Ahmad Al-Shatti, Ahmad Al-Khayat

**Affiliations:** 1 Crisis Decision Support Program, Environment and Life Sciences Research Center, Kuwait Institute for Scientific Research, P.O. Box 24885, 13109 Safat, Kuwait; adabbous@kisr.edu.kw; 2 Faculty of Public Health, Health Sciences Center, Kuwait University, P.O. Box 24923, 13110 Hawalli, Kuwait; janvier.gasana@HSC.EDU.KW; 3 Occupational Health Department, Kuwait Ministry of Health, P.O. Box 51360, 53454 Riqqa, Kuwait; ahmad.alshatti2011@yahoo.com; 4 Techno-Economics Division, Kuwait Institute for Scientific Research, P.O. Box 24885, 13109 Safat, Kuwait; akhayat@kisr.edu.kw

**Keywords:** AirQ+, burden of disease (BOD), DALYs, PM_2.5_, postneonatal mortality, YLD, YLL

## Abstract

Ambient air pollution in terms of fine and coarse particulate matter (PM_2.5_ and PM_10_) has been shown to increase adult and infant mortalities. Most studies have estimated the risk of mortalities through attributable proportions and number of excess cases with no reference to the time lost due to premature mortalities. Disability adjusted life years (DALYs) are necessary to measure the health impact of Ambient particulate matter (PM) over time. In this study, we used life-tables for three years (2014–2016) to estimate the years of life lost (YLL), a main component of DALYs, for adult mortalities (age 30+ years) and postneonatal infant mortalities (age 28+ days–1 year) associated with PM_2.5_ exposure and PM_10_ exposure, respectively. The annual average of PM_2.5_ and PM_10_ concentrations were recorded as 87.9 μg/m^3^ and 167.5 μg/m^3^, which are 8 times greater than the World Health Organization (WHO) air quality guidelines of 10 μg/m^3^ and 20 μg/m^3^, respectively. Results indicated a total of 252.18 (95% CI: 170.69–322.92) YLL for all ages with an increase of 27,474.61 (95% CI: 18,483.02–35,370.58) YLL over 10 years. The expected life remaining (ELR) calculations showed that 30- and 65-year-old persons would gain 2.34 years and 1.93 years, respectively if the current PM_2.5_ exposure levels were reduced to the WHO interim targets (IT-1 = 35 μg/m^3^). Newborns and 1-year old children may live 79.81 and 78.94 years, respectively with an increase in average life expectancy of 2.65 years if the WHO PM_10_ interim targets were met (IT-1 = 70 μg/m^3^). Sensitivity analyses for YLL were carried out for the years 2015, 2025, and 2045 and showed that the years of life would increase significantly for age groups between 30 and 85. Life expectancy, especially for the elderly (≥60 years), would increase at higher rates if PM_2.5_ levels were reduced further. This study can be helpful for the assessment of poor air quality represented by PM_2.5_ and PM_10_ exposures in causing premature adult mortalities and postneonatal infant mortalities in developing countries with high ambient air pollution. Information in this article adds insights to the sustainable development goals (SDG 3.9.1 and 11.6.2) related to the reduction of mortality rates attributed to ambient air levels of coarse and fine particulate matter.

## 1. Introduction

Ambient particulate matter (PM) pollution has been associated with increased risk of premature adult mortalities [1,2,3,4,5] and postneonatal infant mortalities [6,7,8,9,10]. The largest cohort study, the American Cancer Society (ACS) study [11], reported a robust association between long-term exposure to fine inhalable particles (PM_2.5_) and adult mortality (age ≥ 30 years). The Harvard Six-Cities study also found a strong association between long-term exposure to PM_2.5_ and adult mortality, even after controlling for potential confounders, such as age, sex, or cigarette smoking [12]. Both the ACS and the Harvard studies were large prospective cohort investigations with approximately 500,000 and 8111 adults, respectively, followed up for a total duration of 26 years and 15 years, respectively, in order to explore the mortality rates associated with PM_2.5_ exposure after controlling for age, gender, education level, diet, body-mass index, cigarette smoking and occupational exposure. Both studies concluded that PM_2.5_ was positively related to daily mortality, particularly the elderly. Other recent studies have shown similar findings [2,13,14,15,16,17,18]. PM_2.5_ was ranked as the ninth leading risk factor with 3.1 million deaths and 3.1% of global disability adjusted life years (DALYs) in the 2010 global burden of disease (GBD) study [19,20]. In 2015 GBD, PM_2.5_ was the fifth-ranking mortality with 4.2 million deaths and 103.1 million DALYs, representing 7.6% of total global deaths and 4.2% of global DALYs [21]. PM_10_ was also shown to increase the risk of mortality, but mostly to children and infants because of their higher susceptibilities to coarse particles [8,22,23]. Postneonatal mortality (after 28 days of life) is thought to be influenced by the infant’s external environment [24,25].

Within the health risk assessment literature, neither can the probability of exceeding the air standard provide a conclusive evidence on the occurrence of detrimental health effects [26], nor can the incidence of health risk account for the severity and duration of the health impact [27]; therefore, the use of other internationally mortality metrics that address the human burden of disease (BOD) over the years are necessary [28,29]. One of the recently used common metrics is DALYs, which is particularly encompassing since, in addition to the number of deaths, it combines the estimation of time lived with disability and time lost due to premature mortality [30,31,32,33]. There are many applications for the use of DALYs in the assessment of the BOD; including airborne sources [20,26,34], foodborne sources [35,36,37], and waterborne sources [36,38,39]. DALYs values have been continuously updated [40], and improvement of DALYs measures can have a positive impact on the quality of life [41]. Calculation and reporting of disease burdens using DALYs tools have become a routine task of the World Health Organization (WHO) and will continue until 2020 [27,42].

In 2016, the WHO developed the AirQ+ model, updated from the 2014 AirQ2.2 version, to calculate the health burden associated with exposure to the most relevant air pollutants, including PM_2.5_, PM_10_, ozone, nitrogen dioxide, and black carbon. Many studies across the world have used the AirQ+/AirQ2.2 model to quantify the BOD, including Italy [43,44], Poland [45], Greece [46], Estonia [47], Egypt [48], Iran [49,50,51,52,53,54,55], Saudi Arabia [56], and South Korea [57]. Although most of these studies quantified the health burden based on risk estimates, only three studies have used the life-tables approach to estimate the components of DALYs [43,47,49].

In the current study, the life-table approach using the AirQ+ model was used to estimate both premature adult mortalities associated with PM_2.5_ exposure and postneonatal infant mortalities associated with PM_10_ exposure for three years (2014–2016). Mortalities are estimated using the Years of Life Lost (YLL), a main component in the DALYs equation.

## 2. Methods

### 2.1. Air Quality Data

**Figure 1:**
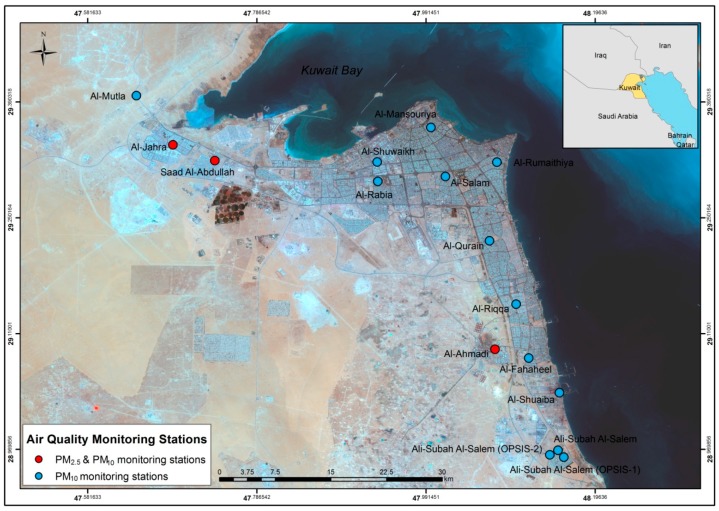
Map showing the location of the three PM_2.5_ monitoring stations in Kuwait (marked in red dots).

The urban districts in Kuwait are grouped into six governates for city planning and municipality services. Altogether, there are a total of 92 urban districts located within the six governates. There are a total of 16 fixed ambient air quality monitoring stations across Kuwait. All sixteen stations record PM_10_ concentrations, while only three stations record PM_2.5_ concentrations (Figure 1).

The study examined the daily data of real-time hourly concentrations of PM_2.5_ and PM_10_ for three years (2014–2016) in association with the collected baseline health data and life tables from the National Center for Health Information (NCHI) of Kuwait Ministry of Health. Careful filtration and processing of monitoring stations’ data was carried out as per the Aphekom project [58]. Stations were excluded if any of the following three conditions existed: (1) more than 75% of missing data in any station; (2) the interquartile range of any station did not overlap with the other stations; (3) the correlation coefficients between stations were lower than 0.6. A fourth condition was added for reconfirmation, that the general acceptable ratio of PM_2.5_/PM_10_ should be within 0.5–0.65 [59,60] or 0.35–0.5 for desert arid regions [59,61].

### 2.2. Life Tables and DALYs

Life tables including mid-year population and deaths by age groups were collected for three years (2014–2016) from the NCHI of Kuwait Ministry of Health. The Health and Vital Statistics Division of the NCHI reports annual health indicators in nine parts, including: demographics, socioeconomics, risk factors, health expenditure, health system, health coverage, health status, selected morbidity, and environmental indicators. Total population data were included in the first part (demographic indicator). Mortality and morbidity rates were included in the seventh part (health status indicator). A total of 18 age ranges were divided in into 5-year intervals from age ≤ 4 years to age ≥ 85 years. The YLL were estimated as a consequence of the impact of PM_2.5_ exposure on population mortality. The YLL is one of the two components in the DALYs equation, in addition to the years lived with disability (YLD). The YLL is a measure of the years lost through premature mortality, while the YLD is a measure of the lost years of healthy life through living in states of less than full health. The DALYs includes both measures and represents an indicator of life expectancy, combining both mortality and morbidity into one summary measure of population health that accounts for the number of years lived in less than optimum health [33]. The basic formulas for calculating DALYs, YLL, and YLD in terms of specific diseases are expressed as [62]:

DALYs = YLL + YLD(1)

YLL = N × L(2)

YLD = I × D × L(3)

where N: number of premature deaths; L: standard life expectancy at age of death (years) in Equation (2) or average duration of disability (years) in Equation (3); I: incident cases (i.e., disabilities); and D: disability weight.

The life table evaluation was conducted using the all (natural) cause mortality for adults (age 30+ years) and postneonatal mortalities (age 28+ days–1 year) as the health endpoints. The total Kuwaiti population for 2014, 2015, and 2016 were estimated as 1,258,254, 1,291,401, and 1,321,593, respectively, as per the consensus data from the Kuwait national statistical office.

### 2.3. AirQ+ Model

This study used recently available AirQ+ software (WHO Regional Office for Europe, Bonn, Germany) (2016), which was designed to quantify the health impact of exposure to air pollution. The tool was developed by the World Health Organization (WHO) regional office for Europe (Bonn office, Germany) to estimate the potential health effects of varied pollutants, including PM_2.5_, PM_10_, NO_2_, O_3_, and black carbon. The AirQ+/AirQ2.2 model was suggested as a reliable tool to estimate the detrimental health effects associated with air pollutants [63] and has been applied internationally to quantify the BOD (Table 1). The AirQ+ model estimates the attributable proportion (AP) of mortalities, the attributable excess incidence per 100,000 (EI), the attributable excess cases, and the YLL after inserting the calculation parameters and the life tables. The calculation parameters inserted into the model include the concentration cut-off value, PM mean concentration, health endpoint baseline incidence and the relative risk (RR). Because of to the high PM concentration levels in Kuwait, the AirQ+ technical officer of the WHO (personal communication) recommended the use of the linear-log RR Equation (4):

RR_lin-log_(C) = expβ[ln(C + 1) − ln(C_0_ + 1)] (4)

AP = (RR − 1)/RR(5)

EI = AP × BI(6)

EC = EI × N(7)

where β is the empirical parameter that denotes the change in the relative risk for one-unit change in the concentration C [64]. C denotes the annual mean pollutant concentration, and C_0_ denotes the cut-off or counterfactual concentration below which one chooses not to quantify the health impact because minimal significant effect on survival has been observed. The cut-off values were selected based on the WHO interim target levels IT-1 as 35 μg/m^3^ and 70 μg/m^3^ for PM_2.5_ and PM_10_, respectively. AP is the attributable proportion (impact fraction): the fraction (percentage) of the health outcome (i.e., postneonatal infant mortality) attributed to the PM_10_ exposure; EI is the excess incidence: the estimated number of attributable cases per 100,000 population at risk; BI is the baseline incidence: the population incidence of the given health effect per 100,000; EC is the excess cases (i.e., the expected number of excess deaths attributed to PM_10_ exposure); N is the relevant exposed population for the health effect. Health endpoint parameters used in this work are summarized in Table 2.

## 3. Results

### 3.1. Baseline Health Data and PM Data

The collected baseline health data and life tables from the NCHI of Kuwait Ministry of Health were examined in relation to the average daily data of PM_2.5_ and PM_10_ for three years (2014–2016). Application of the Aphekom data scrutiny method [58] revealed that 2015 was the only reliable year with an adequate record of air quality data of PM_2.5_ (Al-Ahmadi station) and PM_10_ (Al-Shuwaikh station, Al-Mansouriya station, Al-Salam station, Al-Fahaheel station). A goodness-of-fit test was carried out to determine whether the baseline health data were significantly different between the three years (2014–2016). Table 3 shows the adult and postneonatal mortalities for the three years. The chi-squared test showed no significant differences in mortality cases between the three years (χ^2^ = 0.913, *p* > 0.05, Sig = 0.633). Accordingly, the baseline health data and life tables were analyzed for the year 2015 only. As shown in Table 2, the annual average PM_2.5_ and PM_10_ concentrations for 2015 were 87.9 μg/m^3^ and 167.5 μg/m^3^, respectively which are 8 times greater than the WHO Air Quality Guidelines (PM_2.5_ = 10 μg/m^3^ and PM_10_ = 20 μg/m^3^) [5].

### 3.2. Premature Adult Mortality

**Figure 2:**
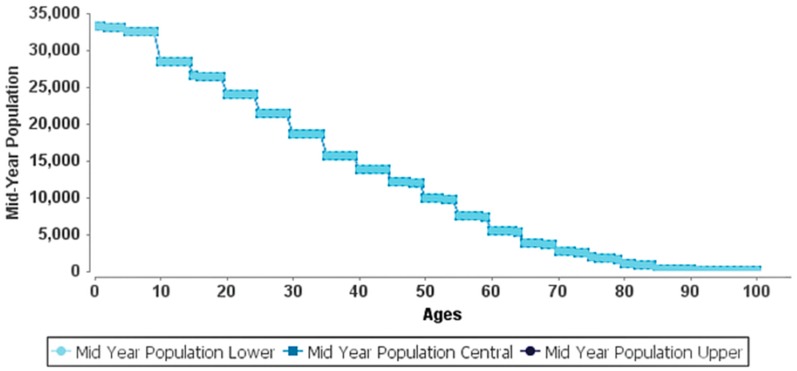
AirQ+ life table evaluation for the year 2015 at the measured PM_2.5_ level (87.9 μg/m^3^).

**Figure 3:**
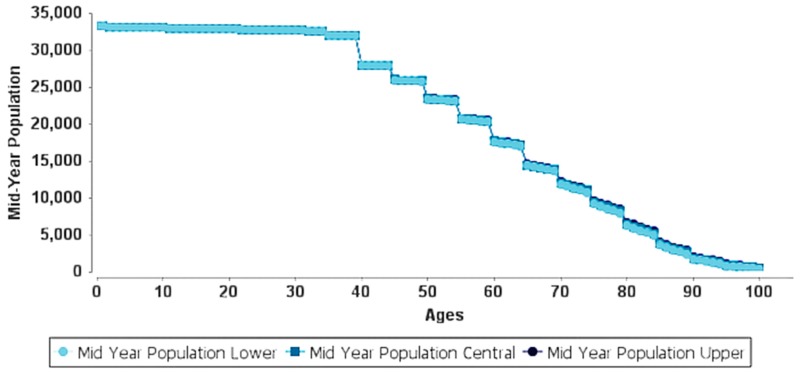
AirQ+ life table evaluation for the year 2045 (30-year forecast) if PM_2.5_ is reduced to the cut-off level (35 μg/m^3^).

Table 4 presents the YLL due to premature mortality attributed to the long-term exposure of PM_2.5_ of 87.9 μg/m^3^. Results indicate that during 2015 there was a total of 252.18 (95% CI: 170.69–322.92) YLL for all ages and 89.53 (95% CI: 60.60–114.64) YLL for ages 0–64. Over ten years (2025), the results showed an increase of 27,474.61 (95% CI: 18,483.02–35,370.58) YLL for all ages and 8487.28 (95% CI: 5741.46–10,873.33) YLL for ages 0–64. Table 5 shows the expected life remaining (ELR) for different age groups: ages 0 and 1 for postneonatal, and ages 30 to 85 for adults. The delta ELR presents the difference between the ELR at the cut-off level (35 μg/m^3^) and the ELR at the measured level (87.9 μg/m^3^) (WHO 2016b). A 30-year-old is expected to live 51.12 more years at the current PM_2.5_ exposure. If the exposure were reduced to the cut-off level, the 30-year-old would gain 2.34 years (95% CI: 1.53–3.08). Similarly, the life expectancy of a 65-year-old may increase by 1.93 years (95% CI: 1.26–2.56) above his/her ELR of 19.02 years if the PM_2.5_ exposure were reduced to the cut-off level. Table 5 also shows that the survival probabilities have a linear relationship with hazard rates; an increase in one results in the decrease of the other with the sum of the two equal to one. Figure 2 and Figure 3 show the AirQ+ life table evaluation for the year 2015 at the measured PM_2.5_ level (87.9 μg/m^3^) and year 2045 (30-year forecast) at the reduced cut-off level (35 μg/m^3^), respectively. For instance, the population at age 30 in 2015 was 18,576 with 18,571 years of life (0.07% hazard rate and survival probability of 99.93%); this number increases to 32,633 in 2045 with 32,624 years of life (0.06% hazard rate and survival probability of 99.94%). The years of life and survival probabilities increase with increasing age; for instance, in 2045, it is forecasted that at age 65, the years of life increase from 14,102 (1.54% hazard rate and survival probability of 98.46%) to 14,413 (1.23% hazard rate and survival probability of 98.77%), a 2% increase in years of life caused by reducing the annual mean concentration of PM_2.5_ from 87.9 μg/m^3^ to the cut-off value of 35 μg/m^3^. Figure 3 shows that the mid-year population after age 40 decreases exponentially, suggesting that the years of life decrease with increasing adult age reaching a minimum at ages ≥ 85.

### 3.3. Postneonatal Infant Mortality

The total infant population was estimated at 32,062 during 2015 in Kuwait. A total of 76 postneonatal infant mortalities were recorded with the probability of occurrence, i.e., the baseline incidence of 237.04 per 100,000. The ELR and other parameters for postneonatal infant mortalities (for age groups 0 and 1) were shown previously in Table 5. The estimated numbers of years that newborns and one-year old children are expected to live are 79.81 and 78.94 years, respectively. A total of 2.65 years (95% CI: 1.35–4.51) and 2.66 years (95% CI: 1.35–4.52) are gained for newborns and 1-year-old children, respectively, if the PM_10_ value was lowered to the WHO IT-1 target (70 μg/m^3^) from the current exposure level of 167.5 μg/m^3^. It is worth noting that for a newborn, the ELR is identical to the life expectancy at birth. The entry population at age 1 was estimated as 33,215 with years of life approximation at 33,189, corresponding to a survival probability of 99.84% at a hazard rate of 0.16%.

Table 6 presents the attributable proportion (AP), excess cases, and excess incidence (per 100,000 at risk) for the postneonatal infant mortality associated with the PM_10_ exposure of 167.5 μg/m^3^. The AP of postneonatal mortality due to PM_10_ exposure was estimated at 22.68% (95% CI: 12.18–35.84). A total of 17 excess death cases were attributed to the PM_10_ exposure, which is equivalent to an excess incidence of 53.77 per 100,000 (95% CI: 28.87–84.95). The relative risk (RR) was calculated as 1.293; that is with every 70 μg/m^3^ increase in annual average PM_10_ concentration, the associated postneonatal infant mortality increased by 29.3%.

### 3.4. Sensitivity Analysis

**Figure 4:**
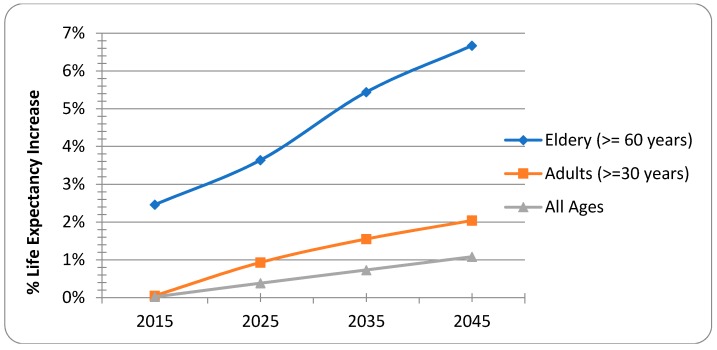
Sensitivity analysis for % of life expectancy for the years 2015, 2025, 2035, 2045.

Results of the sensitivity analysis for YLL and premature mortality for different age groups are shown in Table 7 and Figure 4. For all ages, reduction of the PM_2.5_ exposure from the current level (87.9 μg/m^3^) to the cut-off level (35 μg/m^3^) would increase the years of life from 2,088,845 years to 2,111,318 years (1.08%) after a 30-year forecast (year 2045). However, when considering only the adults ≥ 30 years, the years of life would increase by 2.04% from 1,101,798 years to 1,124,271 years. The chi-squared test for adults ≥ 30 years showed that the years of life are significantly different between the current and the cut-off levels, indicating that life expectancy for older adults would increase more by the reduction of PM_2.5_ exposure levels (χ^2^ = 71.327, *p* ≤ 0.01, Sig = 0.000). Moreover, the years of life would increase by 6.67% from 294,436 years to 314,091 years for the elderly (≥60 years).

## 4. Discussion

DALYs’s use in the quantification of the BOD is a systematic, scientific technique to estimate the comparative magnitude of health loss due to diseases and risk factors by age for specific points in time. DALYs’s use in estimating the health loss can be measured through premature deaths (YLL) and/or non-fetal disability (YLD). This study estimated only the first component in the DALYs equation, which is the YLL. The standard life tables received from the NCHI of Kuwait Ministry of Health were confined to compute the YLL at each age. The nature of life tables obtained did not enable us to measure the YLD or perform any age-sex group comparison. The AirQ+ model used in this study did not discount the YLLs for time or age. The assumption was made to treat a year of healthy life as equal irrespective of the age at which it is lived [20]. This assumption was shown to be conceptually and scientifically correct [27,29] because age-weights allow for bias in valuing life years in different age ranges [65,66] and time-weights distinguish the relative value of healthy life lost in different time periods [67]. Time and age discount-drop in the calculations of YLL were also applied in similar studies [43,47,49,68,69]. DALYs methodology using the life-table approach has been adopted by the WHO Global Burden of Disease as a standardized method for ease of comparison of life losses across countries. Life-table parameters, including age, gender, mortality counts, incidence counts, population counts, disease duration, and disability weights are needed in order to calculate the DALYs. The strength of using the life-table approach is the provision of the overall measure of mortality and morbidity, allowing countries and regions to be compared and therefore provides a comparative assessment of the mortality rates and ill-health effects of a certain population’s lifetime.

The YLL of premature mortalities for 2015 and for the next 10 years were estimated at 252.18 and 27,474.61, respectively assuming that the annual average PM_2.5_ concentration (87.9 μg/m^3^) does not change over this period. The YLL estimated for Kuwait was lower than the YLL estimated for Northern Italy (433 YLL in 2007) [43], Tallinn, Estonia (3859 YLL in 2006) [47], and Tehran, Iran (67, 970 YLL in 2015) [49]. Scaling these numbers to the rate per 100,000 residents revealed that Kuwait was much lower YLL (19.5) relative to Northern Italy (2586), Tallinn (988), and Tehran (786), especially when taking into account its higher PM_2.5_ concentration (87.9 μg/m^3^) relative to Northern Italy (42 μg/m^3^), Tallinn (11.6 μg/m^3^) and Tehran (26 μg/m^3^). Although the reference years of Northern Italy and Tallinn are not directly comparable to Kuwait’s reference year, the YLL calculated provides information on the consistency of the results obtained by the AirQ+ model. This study also showed that at the current concentration level, the average life expectancy of a 30-year-old person is reduced by more than 2 years, while the average decrease in life expectancy at birth was roughly 3 years, corresponding to an AP of 22.68% and 17 excess cases (53.77 per 100,000). Pope (2000) reported that the US population as a whole loses 1–3 years of life due to PM_2.5_ exposure. A study by Boldo, Medina [70] of 23 European cities reported that the average life expectancy at birth would increase more than 2 years if PM_2.5_ exposure was reduced in heavily polluted cities, such as Bucharest and Rome. In a WHO report, the average life expectancy at birth from all European Union populations was estimated to be shortened by 8.6 months due to PM_2.5_ exposure, and with proper air quality control measures, the average life expectancy would increase by 2.3 months by 2020, equating to an annual avoidance of one million YLL [47].

Sensitivity analysis showed that reduction of the current PM_2.5_ level to the WHO IT-1 interim target of 35 μg/m^3^ would increase life expectancy and reduce the hazard rate of premature adult mortality from 0.07–10.58% to 0.06–8.47% for age groups between 30 and 85 for the year 2045. Negative health endpoints were shown to be associated with high-risk groups, such as people with pre-diagnosed respiratory or cardiovascular diseases or the elderly [71]. Kuwait’s older populations with high background rates of cardiovascular diseases, i.e., ischemic heart disease and stroke, may have contributed to the higher YLL rate at older ages [72]. Regions with older-than-average populations and high underlying rates of cardiovascular disease (e.g., the former Soviet Union) tend to have higher rates of attributable mortalities at higher ages [13].

Without underrating the postneonatal infant mortalities from exposure to coarse particles (PM_10_), only part of the total BOD is captured since the YLLs are generally unknown. Short-term effects of PM are only one-tenth to one-fifth of the overall effect from long-term exposure [73]; therefore, the long-term exposure estimates of PM_2.5_ are better indicators of premature mortalities and DALYs. Avoiding death from a PM_2.5_ exposure does not necessarily mean saving a life; however, PM_2.5_ exposure substantially reduces life expectancy [74].

The AirQ+ model was selected to quantify the BOD associated with premature adult mortalities due to PM_2.5_ exposure and postneonatal infant mortalities due to PM_10_ exposure in terms of the YLL using life tables, but it has limitations. The model does not allow for the calculation of the YLD which is considered important to properly evaluate the DALYs for a comprehensive health impact assessment in terms of time lived with disability. The model’s long-term effects of PM are calculated based on risk estimates from cohort studies carried out in the USA and Europe [11,59,75], where concentrations are low and consequently, may overestimate the BOD in hot arid environments with higher PM concentrations, such as Kuwait, given the high intensity and frequency of dust storms [76,77,78,79]. However, based on the recommendations of the WHO technical officer (personal communication) the use of the linear-log calculation method would offset such overestimation. The AirQ+/AirQ2.2 model was shown to be a valid and reliable tool to estimate the potential health impacts from short-term exposures [44,63]. More research is needed on the AirQ+ model to enhance the calculation of health outcome from long-term exposures [80].

Future studies are needed to estimate the age-sex-geography health impacts of DALYs in Kuwait. No health assessment studies using the AirQ+ models of life tables were previously conducted to estimate the combination of age-sex-geography risk. Studies with longer time scale durations are required. Other DALYs from water-borne or food-borne exposures should be also investigated [81,82], however, the YLLs and YLDs from these diseases may not be critical for Kuwait given the high standards of living and the provision of adequate sanitary and food safety measures.

## Figures and Tables

**Table 1:** International use of the AirQ+/AirQ2.2 model.

Country *	PM_2.5_	PM_10_	AP	YLL	Reference
Egypt	√		√		Wheida et al., 2018
Estonia	√	√	√	√	Orru et al., 2009 **
23 European Cities	√	√	√		Boldo et al., 2006
Greece		√	√		Moustris et al., 2017
Iran	√	√	√	√	Faridi et al., 2018 **; Ghozikali et al., 2016; Goudarzi et al., 2017; Hadei et al., 2018; Hopke et al., 2018; Khaniabadi et al., 2018; Miri et al., 2017
Italy	√	√	√	√	Fattore et al., 2011 **; Tominz et al., 2005
Poland		√	√		Skotak and Swiatczak, 2008
Saudi Arabia		√	√		Habeebullah, 2013
South Korea		√	√		Jeong, 2013

* In alphabetical order; ** Study estimated both the AP and YLL. AP: Attributable Proportion: The fraction of the health outcome (i.e., postneonatal infant mortality) attributed to the PM_10_ exposure. YLL: Years of Life Lost.

**Table 2:** Health endpoints used in the AirQ+ model in this study.

Health Endpoint	Exposure	Cut-off Value (μg/m^3^)	Annual Mean (μg/m^3^)	RR *	β Coefficient *
Premature adult mortality, all causes	PM_2.5_	35	87.9	1.062 (1.04,1.083)	0.2454 (0.1600, 0.3252)
Postneonatal infant mortality, all causes	PM_10_	70	167.5	1.04 (1.02, 1.07)	0.2976 (0.1502, 0.5134)

* Values in parentheses are lower and upper bounds (95% CI); RR: Relative Risk.

**Table 3:** Adult and postneonatal mortalities for three years (2014–2016).

Mortality	2014	2015	2016
Adult	2561	2735	2685
Postneonatal	73	76	86

Pearson Chi-Square (χ^2^) = 0.913, df = 2, Sig = 0.633.

**Table 4:** Years of Life Lost (YLL) due to premature mortality.

Measure	Age	Central	Lower	Upper
YLL–2015	all ages	252.18	170.69	322.92
YLL–2015	age 0–64	89.53	60.60	114.64
YLL over 10 Years–2025	all ages	27,474.61	18,483.02	35,370.58
YLL over 10 Years–2025	age 0–64	8487.28	5741.46	10,873.33

Values are written as 95% confidence interval: central (lower–upper).

**Table 5:** Expected Life Remaining (ELR) and other parameters for different age groups.

Age	ELR (years)	Delta ELR *	Entry Population	Years of Life	Hazard Rate	Survival Probability
0	79.81	2.65 (1.35, 4.51)	33,269	33,242	0.16%	99.84%
1	78.94	2.66 (1.35, 4.52)	33,215	33,189	0.16%	99.84%
30	51.12	2.34 (1.53, 3.08)	18,576	18,571	0.06%	99.94%
35	46.29	2.31 (1.51, 3.05)	15,735	15,730	0.07%	99.93%
40	41.47	2.28 (1.49, 3.01)	13,885	13,878	0.11%	99.89%
45	36.73	2.24 (1.47, 2.96)	12,123	12,113	0.16%	99.84%
50	32.08	2.19 (1.43, 2.89)	9910	9898	0.25%	99.75%
55	27.54	2.12 (1.39, 2.81)	7564	7550	0.38%	99.62%
60	23.14	2.04 (1.33, 2.70)	5561	5542	0.66%	99.34%
65	19.02	1.93 (1.26, 2.56)	3895	3871	1.23%	98.77%
70	15.36	1.78 (1.16, 2.36)	2812	2782	2.16%	97.84%
75	12.25	1.58 (1.03, 2.10)	1939	1907	3.29%	96.71%
80	9.54	1.37 (0.89, 1.81)	1042	1014	5.28%	94.72%
85	7.46	1.10 (0.72, 1.45)	381	364	8.47%	91.53%

* Obtained using the lower and upper estimates of the RR values.

**Table 6:** Postneonatal infant mortality with the associated parameters.

	Central	Lower	Upper
AP *	22.68%	12.18%	35.84%
Excess Cases **	17	9	27
Cases per 100,000 ***	53.77	28.87	84.95

* Attributable Proportion. ** Excess number of cases attributed to PM_10_ exposure. *** Excess incidence (cases per 100,000 at risk).

**Table 7:** Sensitivity analysis for YLL and premature mortality for different age groups.

Age	Years of Life at the Current PM_2.5_				Years of Life at the Cut-Off PM_2.5_			
	2015	2025	2035	2045	2015	2025	2035	2045
0–1	66,431	66,431	66,431	66,431	66,431	66,431	66,431	66,431
2–9	261,498	264,168	264,168	264,168	261,498	264,168	264,168	264,168
10–19	274,269	326,535	329,197	329,197	274,269	326,535	329,197	329,197
20–29	226,802	271,655	324,606	327,251	226,802	271,655	324,606	327,251
30–39	171,239	225,351	270,909	322,526	171,251	225,508	271,099	322,757
40–49	129,500	169,332	222,815	267,848	129,521	169,714	223,473	268,644
50–59	86,543	126,119	164,919	216,988	86,576	126,807	166,172	218,779
60–69	45,971	80,746	117,527	153,736	46,022	81,899	119,836	157,075
70–79	21,891	37,249	65,321	94,749	21,962	38,888	69,098	100,819
≥80	7251	14,884	26,376	45,951	7314	16,929	31,683	56,197

Pearson Chi-Square (χ^2^) (for ages ≥ 30) = 71.327, df = 3, Sig = 0.000. Pearson Chi-Square (χ^2^) (for ages ≥ 60) = 132.10, df = 3, Sig = 0.000.
